# Conceptual Framework to Guide Early Diagnosis Programs for Symptomatic Cancer as Part of Global Cancer Control

**DOI:** 10.1200/GO.20.00310

**Published:** 2021-01-06

**Authors:** Minjoung Monica Koo, Karla Unger-Saldaña, Amos D. Mwaka, Marilys Corbex, Ophira Ginsburg, Fiona M. Walter, Natalia Calanzani, Jennifer Moodley, Greg P. Rubin, Georgios Lyratzopoulos

**Affiliations:** ^1^ Epidemiology of Cancer Healthcare and Outcomes (ECHO) Research Group, Department of Behavioural Science and Health, University College London, London, United Kingdom; ^2^ CONACYT (National Council of Science and Technology)–National Cancer Institute, Mexico City, Mexico; ^3^ Department of Medicine, School of Medicine, College of Health Sciences, Makerere University, Kampala, Uganda; ^4^ WHO Regional Office for Europe, Copenhagen, Denmark; ^5^ Perlmutter Cancer Center and the Department of Population Health, NYU Grossman School of Medicine, NYU Langone Health, New York, NY; ^6^ The Primary Care Unit, Department of Public Health and Primary Care, University of Cambridge, Cambridge, United Kingdom; ^7^ Women's Health Research Unit, School of Public Health and Family Medicine, Faculty of Health Sciences, University of Cape Town, Cape Town, South Africa; ^8^ Cancer Research Initiative, Faculty of Health Sciences, University of Cape Town, Cape Town, South Africa; ^9^ SAMRC Gynaecology Cancer Research Centre, Faculty of Health Sciences, University of Cape Town, Cape Town, South Africa; ^10^ Institute of Health and Society, Newcastle University, Sir James Spence Institute, Royal Victoria Infirmary, Newcastle upon Tyne, United Kingdom

## Abstract

Diagnosing cancer earlier can enable timely treatment and optimize outcomes. Worldwide, national cancer control plans increasingly encompass early diagnosis programs for symptomatic patients, commonly comprising awareness campaigns to encourage prompt help-seeking for possible cancer symptoms and health system policies to support prompt diagnostic assessment and access to treatment. By their nature, early diagnosis programs involve complex public health interventions aiming to address unmet health needs by acting on patient, clinical, and system factors. However, there is uncertainty regarding how to optimize the design and evaluation of such interventions. We propose that decisions about early diagnosis programs should consider four interrelated components: first, the conduct of a needs assessment (based on cancer-site–specific statistics) to identify the cancers that may benefit most from early diagnosis in the target population; second, the consideration of symptom epidemiology to inform prioritization within an intervention; third, the identification of factors influencing prompt help-seeking at individual and system level to support the design and evaluation of interventions; and finally, the evaluation of factors influencing the health systems’ capacity to promptly assess patients. This conceptual framework can be used by public health researchers and policy makers to identify the greatest evidence gaps and guide the design and evaluation of local early diagnosis programs as part of broader cancer control strategies.

## INTRODUCTION

Cancer control is a global health priority.^1^ In high-income countries (HICs), cancer incidence is increasing with other noncommunicable diseases, whereas in lower- and middle-income countries (LMICs), there is often a concordant double burden of communicable diseases.^2^ Furthermore, prolonged time to diagnosis and treatment is disproportionately experienced by poorer individuals, contributing to disparities in cancer outcomes within and between countries.^1,3^

**CONTEXT**

**Key Objective**
Prompt diagnosis and treatment of symptomatic patients can improve cancer outcomes, but this depends on many individual and health system factors. What are the key considerations that need to be taken into account when developing early diagnosis programs?
**Knowledge Generated**
The design, implementation, and evaluation of early diagnosis programs should be informed by the assessment of population-specific health needs; the epidemiology of symptoms of possible cancer; and the consideration of individual and health system factors influencing both timely help-seeking for new symptoms and timely investigation and treatment. Framed within the broader evidence base, case studies from the United Kingdom, Denmark, Malaysia, and Zambia are used to illustrate existing early diagnosis activities.
**Relevance**
This framework can help identify population-specific evidence gaps in early cancer diagnosis research and guide the design and evaluation of early diagnosis programs across the globe.

Diagnosing symptomatic cancer earlier is a feasible and cost-effective strategy^4,5^ that can contribute to better clinical outcomes^6-9^ and improve patient experience.^10-12^ Effective asymptomatic detection is currently only available for a few cancers, and even in countries with established population-based screening programs, the majority of patients with cancer are diagnosed following symptomatic presentation.^3,13,14^ Furthermore, in low-resource settings where screening programs are not available or feasible,^15-17^ early diagnosis (also known as clinical downstaging) strategies can support their introduction by improving clinical pathways and building diagnostic capacity.^18^

Early diagnosis programs consist of supporting prompt help-seeking among symptomatic individuals and/or enabling timely access to diagnosis and treatment.^1^ Public education campaigns aiming to raise awareness of cancer and its symptoms and signs among the general population have been conducted in both HICs and LMICs (Box 1)^19,26,29-33^, whereas fast-track pathways and clinical guidelines aiming to expedite the investigation, diagnosis, and treatment of symptomatic individuals have been introduced in HICs including the United Kingdom, Denmark, New Zealand, and Spain (Box 2).^39,45-48^

BOX 1. Examples of Symptom Awareness Campaign Design and Evaluation in England and Malaysia

In England, national Be Clear on Cancer campaigns were developed in 2012 through the partnership of governmental agencies and Cancer Research UK, a charity. Nationwide campaigns designed to target individuals of age 50+ years have been conducted for colorectal, lung, breast, bladder, kidney, and esophagogastric cancers.^19,20,25^ The visual and linguistic components of the campaign were designed to engage individuals from lower socioeconomic groups and alleviate possible concerns about bothering the doctor.^21^ Campaign evaluations have found increasing levels of awareness and frequency of help-seeking for some symptoms and increased referrals and use of investigations for suspected cancer by primary care physicians although evidence of improvement in stage at diagnosis and survival is limited.^22-25^

In Malaysia, breast and colorectal cancers were the focus of Be Cancer Alert, a culturally sensitive mass media campaign led by a multisector partnership of researchers from Malaysia and the United Kingdom.^26^ The campaign focused on breast and colorectal cancers because of the high prevalence of these cancers in Malaysia, and the design was driven by evidence suggesting low awareness of the alarm symptoms of breast and colorectal cancer among members of the public as well as negative attitudes and beliefs regarding cancer. The campaign's logic model takes contextual factors into account, including differences in knowledge and cultural norms between the main ethnic groups in Malaysia (Malays, Chinese, and Indian) and health and media literacy.^27^ Recently published findings indicate improvements in symptom awareness at follow-up across ethnic and social strata although, in common with the English experience, evidence of improvements in downstream outcomes was less clear.^28^

BOX 2. Fast-Track Pathways for Suspected Cancer in England and Denmark

For most common cancers, cancer survival in England (United Kingdom) and Denmark is comparatively poor to other high-income countries.^34,35^ This has stimulated the relative prioritization of early diagnosis as part of cancer control measures in these countries.

Fast-track (2-week-wait) referral pathways from primary care to specialist assessment were introduced in England in 1999/2000.^36^ In parallel, national clinical guidelines have been developed to support the pathways: certain red-flag alarm symptoms are recommended for referral based on their positive predictive value for cancer.^37^ Around 2.4 million symptomatic individuals are referred using this pathway annually, of which 93% occur within 14 days and around 8% are subsequently diagnosed with cancer.^36,38^ For individuals subsequently diagnosed with cancer (through any route), national targets for the treatment interval (time from decision to treat to first treatment) are set at a maximum of 31 days.^38^ In Denmark, a similar fast-track referral system was introduced in 2008 called the Cancer Patient Pathway.^39^

Evaluating the impact of the fast-track pathways is challenging because of the waiting time paradox, whereby individuals with more severe and acute onset symptoms are diagnosed quickly but have poorer survival compared with patients who experience longer time to diagnosis.^6,7^ Accounting for this phenomenon, the introduction of fast-track pathways has been associated with improvements in cancer survival.^40,41^ Primary care practices with higher usage of the 2-week-wait referral pathway have been associated with lower mortality in England.^42^ Furthermore, increasing use of the 2-week-wait pathway over time has been associated with decreasing proportions of patients diagnosed with cancer as an emergency,^43^ a marker of poor prognosis.^44^

## THE NEED FOR A FRAMEWORK TO GUIDE DECISION MAKING

The timeliness of cancer diagnosis and treatment in symptomatic patients has been conceptualized as a series of intervals starting from symptom onset.^12,49-51^ Building on these theoretical foundations, it is important to consider how early diagnosis evidence can be translated into interventions.

Early diagnosis programs represent complex interventions; they aim to achieve changes in population-level behavior and often interdependent health system factors.^52,53^ The majority of early diagnosis initiatives are implemented as natural experiments,^22,54,55^ compounding the difficulties in measuring impact and effectiveness.

**FIG 1:**
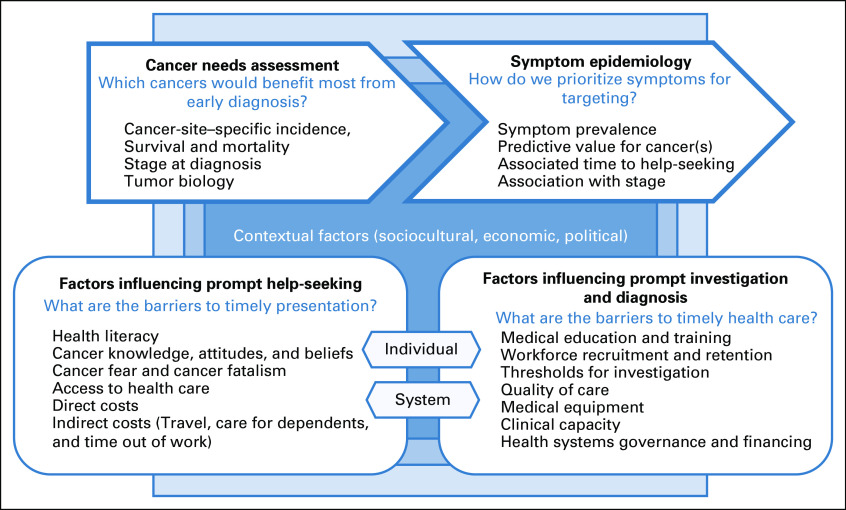
Conceptual framework for the design and evaluation of early diagnosis programs for cancer.

We propose a framework to aid the design and evaluation of early diagnosis programs (Fig 1). It draws on existing literature from HIC and LMIC settings, augmented by the expert opinions of the international group of authors. The framework comprises four components: first, the conduct of a needs assessment (based on cancer-site–specific statistics) to identify the cancers that may benefit most from early diagnosis in the target population; second, the consideration of symptom epidemiology to inform prioritization within an intervention; third, the identification of factors influencing prompt help-seeking at individual and system level to support the design and evaluation of interventions; and finally, the appraisal of factors influencing the health systems’ capacity to promptly assess patients.

## CANCER NEEDS ASSESSMENT

Local or contextualized epidemiological knowledge about the incidence, mortality, and survival associated with different cancers in a given setting can inform the prioritization of cancer types when designing an early diagnosis program. These considerations should ideally be based on high-quality population-based cancer registries and mortality data, although this is conditional on cancer registry infrastructure.^56^ Cancer-specific incidence alone may be instructive, whereas other characteristics such as the frequency and distribution of advanced stage at diagnosis among cases, site- and stage-specific survival statistics, and existing levels of awareness among the general population can further contribute to the prioritization of cancer types to be targeted by early diagnosis programs.^12^ In the absence of such data, as in many LMICs, data collection may be necessary to gauge capacity for early diagnosis; local- or regional-level statistics from hospital-based cancer registries or other sources could be useful. Contextual factors such as stakeholder views, sociocultural and political factors, and health system factors such as access and affordability of cancer treatment and healthcare providers will also influence how cancers are ranked by the need for early diagnosis^57^ and may often be stronger drivers for action particularly in settings where data capture and collection are difficult.

## SYMPTOM EPIDEMIOLOGY

Early diagnosis interventions are, by their nature, centered on specific presenting symptoms of cancer. Accordingly, understanding symptom epidemiology, that is, population-level evidence regarding the nature and frequency of presenting symptoms, can help the targeting of interventions.^58^

Evidence on presenting symptoms may be collected retrospectively through patient recall or extracted from health records where information is captured prospectively during consultations. Self-report-based approaches provide firsthand insight, although may be associated with recall bias (because of the retrospective nature of recalling experienced symptoms) and survival bias (where patients who are the sickest are less likely to be included). In comparison, prospective health record studies allow for the study of much larger populations, but incomplete symptom elicitation and recording may introduce other biases.^59^ The latter may be used to derive the positive predictive value (PPV) of symptoms for cancer, which expresses the probability that following presentation with a symptom, that individual will be diagnosed with cancer.

There is a large body of literature on symptom-specific PPVs for cancer based on primary care electronic health record studies in England (see Table 1 for selected examples).^60-75^ This evidence informed the development of an explicit PPV threshold of 3% used to prioritize symptoms for urgent investigation and fast-track 2-week-wait referral in England.^36,37^ Currently, there is no available evidence, and no biological reason, to suggest that the nature of cancer symptoms or their relative frequency at presentation may vary between different countries, although psychosocial and health system factors could influence how symptoms are reported.^76,77^ The English studies indicate that PPVs increase with age and are higher among men; other differences in demographic factors, pre-existing conditions (morbidities), and symptom prevalence could lead to variation in symptom PPVs across populations, for example, the PPV of respiratory symptoms for lung cancer in communities with high tuberculosis prevalence. As LMICs develop electronic medical records, examining country-specific symptom prevalence and PPVs will be necessary.

**TABLE 1:**
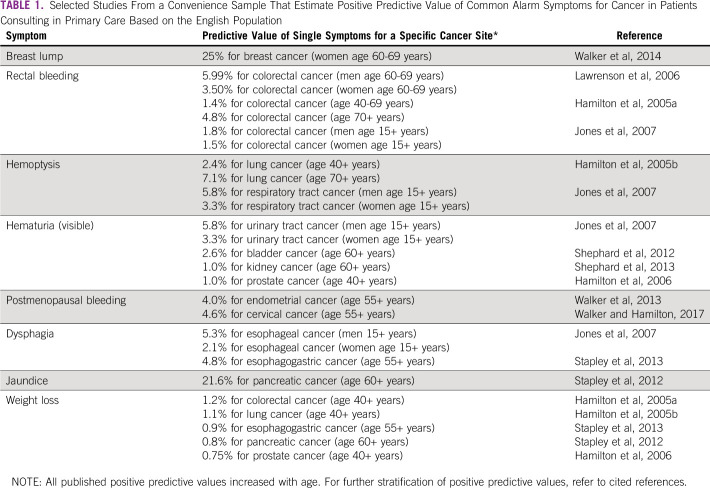
Selected Studies From a Convenience Sample That Estimate Positive Predictive Value of Common Alarm Symptoms for Cancer in Patients Consulting in Primary Care Based on the English Population

Nevertheless, around 50% of patients with cancer present with nonspecific or vague symptoms that are associated with much lower PPVs for cancer.^78^ In Denmark, patients with nonspecific symptoms (eg, unexplained weight loss, fatigue, and abdominal pain) are referred to multidisciplinary clinics where a battery of investigations (blood and urine tests and diagnostic imaging) can be conducted rapidly.^39^ Similarly designed clinics have been introduced in the United Kingdom to accommodate individuals who present with nonalarm symptoms.^79-83^

Evidence regarding pre-existing levels of symptom awareness and symptom-specific measures of diagnostic timeliness could guide the selection of symptoms in early diagnosis programs. Validated measures including the Cancer Awareness Measure,^84,85^ the Awareness and Beliefs about Cancer measure,^86^ and region-specific adaptations such as the African Women Awareness of CANcer tool designed for breast and cervical cancer^87^ could support such prioritization by identifying symptoms correlated with low awareness.^76,88,89^ Symptoms associated with the longest patient intervals (time from symptom onset to presentation) may also be particularly worthy of targeting.^90^

## PROMPT HELP-SEEKING FOR SYMPTOMS

Psychological, social, and cultural factors influence the timeliness of help-seeking for symptoms.^49,51,91,92^ Individual- and group-level (eg, family or social network) barriers include cancer fear and stigma, poor health literacy, lack of trust in healthcare providers, and low expectations or perceptions regarding healthcare access or quality.^3,93-98^ Such barriers have been associated with demonstrably longer intervals to help-seeking for possible cancer symptoms^99,100^ and advanced stage at diagnosis among those diagnosed with cancer.^101,102^

System-wide factors also contribute to delayed presentation and inequalities in cancer outcomes. These include the proximity and accessibility of health care; the availability of infrastructure and transport services; the direct costs of seeking medical advice, diagnostic testing, and anticipated treatment; and the indirect costs of seeking help including travel and accommodation costs, care for dependents, and time out of work.^103-106^ Addressing the individual and system factors influencing prompt help-seeking is important for improving equitable access to cancer services, given that the above barriers are invariably more prevalent in low-resource communities.

## PROMPT INVESTIGATION AND DIAGNOSIS

Many additional steps lie between prompt help-seeking for symptoms, the diagnosis of cancer, and the initiation of treatment. Postpresentational intervals in LMICs often account for a greater proportion of total time to cancer diagnosis and treatment compared with help-seeking intervals.^98,107^ Furthermore, focusing on early diagnosis is an important precursor to establishing cancer screening programs by increasing diagnostic capacity for all individuals with positive results.^108^ Early diagnosis programs have the potential to contribute to health system development as the diagnosis and treatment of cancer requires a coordinated multidisciplinary approach. The consideration of health system factors is therefore critical for early diagnosis programs.^12,109,110^

Timely investigation and diagnosis of cancer relies on the availability of medical equipment, imaging, pathology, and clinical laboratory services. Increasing capacity for diagnostic technologies should be preceded by the consideration of affordability and sustainability within the existing health system infrastructure for the health system and population.^111^ In England, the increasing volume of fast-track referrals (Box 2) has added to pressures on secondary care services such as colonoscopy provision and imaging, pointing to the need for new technologies and interventions to support risk management and clinical decision making, including new service pathways.^79,112^ Fecal immunochemical testing, although initially introduced in the context of bowel cancer screening, is increasingly considered as a sensitive triaging tool for patients presenting with abdominal symptoms to manage demand for colonoscopy services.^113-115^ Access to and availability of timely cancer treatment is also critical for early diagnosis to contribute to cancer outcomes. In Zambia, decentralized models of service provision that connect different levels of care present an effective alternative to comprehensive cancer centers, a common model of care in LMICs, while repurposing existing care pathways (Box 3).^118^

BOX 3. Implementation of Primary Care–Based Breast Cancer Services in Zambia

The majority of breast cancers in Zambia are currently diagnosed at stage III/IV, and early diagnosis has been identified as a priority.^116^ Symptomatic individuals have to navigate multiple levels of the health system before diagnosis and treatment at the national cancer care center in Lusaka. A national assessment of health services for breast and cervical cancer identified the need for better coordination of early detection services for breast cancer.^117^

A breast cancer clinic was established at primary care level in a district hospital.^118^ Symptomatic women from surrounding primary care facilities including rural health posts, health centers, and other district hospitals are assessed at the clinic. Initially, an existing cervical cancer prevention program was used to direct eligible women for assessment and generate awareness of the new service among primary care providers. The clinic provides timely diagnostic investigation (clinical breast examination, same-visit ultrasound, and core- or fine-needle biopsy) and has surgical capacity. Patients requiring chemotherapy or radiation are referred within 2 weeks to the national cancer care center. These decentralized care pathways improved timeliness and access to diagnosis and treatment services by integrating provision with existing care pathways.^118^ The intervention has been accompanied by workforce development, which was identified as a barrier to cancer care delivery in Zambia.^109^

Recruitment and retention of a skilled health workforce including community health workers, specialists, and allied health professionals (such as radiologists, pathologists, and biomedical scientists) are also pivotal. Strengthening knowledge and awareness of cancer signs and symptoms among the healthcare workforce (particularly those working in frontline services) is of equal, if not greater, importance to raising awareness among the general population.^119-123^

Increasing health system capacity for timely cancer diagnosis and treatment relies on macro-level factors including health system governance and financing. Emigration of trained professionals to HICs may reduce their availability in LMICs.^124^ Although strengthening health systems can be challenging, creating or improving clinical pathways for the diagnosis of cancer can provide impetus and opportunities to improve healthcare delivery for other nonmalignant diseases, collinear to the universal healthcare agenda.^108,124,125^

## DESIGNING AND EVALUATING EARLY DIAGNOSIS PROGRAMS: CHALLENGES AND OPPORTUNITIES

Early diagnosis programs comprise complex interventions, both acting on and being influenced by multiple interplaying factors.^12^ The conceptual framework we propose aims to make the rationale of such interventions explicit and in doing so support evidence-based strategies for earlier diagnosis.

## DESIGN AND IMPLEMENTATION

In settings with fragmented or nonexistent data infrastructures, population-specific health needs assessments prior to the design and implementation of early diagnosis programs will require adaptation to available data. Nevertheless, systematically synthesizing up-to-date evidence to inform intervention design remains challenging even where routine data are readily available.^55^ In some instances, primary data collection may be a necessary component, using instruments such as patient questionnaires and clinical audit to identify existing barriers to early diagnosis.^87,119,126^

Incorporating implementation science can help identify mechanisms that enable change or lead to improved outcomes; it also helps to understand the role of contextual influencers.^127,128^ The use of implementation science while also embracing complexity and systems thinking can help to tackle the gap between evidence and current practice.^129-132^ Measuring outcomes specific to implementation can inform how best to adapt an intervention to real-life settings, determine effectiveness, and sustain and scale up efforts.^127,133^ The Behaviour Change Wheel^128^ and Normalization Process Theory^134^ are some of the many theories and frameworks that have been adopted to understand and assess implementation in early cancer diagnosis programs in the United Kingdom, Denmark, and Australia.^135-140^

## MONITORING AND EVALUATION

Monitoring and evaluation are critical to the success of an early diagnosis program.^12^ Early diagnosis programs target changes in population-level behavior or health system improvements, which can be difficult to quantify. For example, symptom awareness campaigns may stimulate transgenerational changes in attitudes toward cancer and contribute to timely diagnosis later in the life course,^141^ whereas health system–wide changes may contribute to broader improvements in healthcare delivery and help streamline diagnostic pathways for cancer. Although some randomized control trials have been conducted,^120,142^ the majority of early diagnosis programs are natural experiments and so evaluations more often take other forms such as before and after designs and interrupted time series analysis, commonly involving mixed-methods research.^22,25,27,40,135,143-145^

Process and outcome indicators for monitoring early diagnosis programs have been recommended by the WHO.^12^ Measures should be selected based on available resources and existing infrastructure, which will influence their relative utility. For example, the proportion of patients with cancer diagnosed at early stage is often the most direct outcome indicator but is contingent on reliable and accurate cancer registration services. The patient interval (time from symptom onset to help-seeking) can be measured to evaluate possible improvements in symptom awareness among patients with cancer; here, audit methodologies or self-reported data may be more informative than health records, which can often be incomplete.^146^

Other metrics may be specific to certain intervention types and health system contexts. For example, a range of process and outcome measures have been examined for the evaluation of the Be Clear on Cancer campaigns in England.^25^ These include the cancer referral rate (the number of urgent referrals for suspected cancer made by primary care practices divided by the registered practice population) and referral scheme sensitivity (the number of cancer diagnoses resulting from an urgent referral, rather than another route to diagnosis). The primary care interval (time from first presentation in primary care to referral to secondary care) has also been highlighted as a measure of diagnostic timeliness in England, Scandinavia, and other countries^50,147^ but is of less relevance to jurisdictions where patients may access specialist care directly.

In conclusion, increasing cancer incidence globally compels public health agencies to instigate cancer prevention and control measures; early diagnosis programs in both HIC and LMIC settings form a critical component. Cancer-specific health needs assessment followed by the consideration of symptom epidemiology can contribute to priority setting within programs. Programs should also consider societal and health system–level factors that influence prompt help-seeking, investigation, and diagnosis of suspected cancer. The framework we propose can help to identify population-specific evidence gaps and optimize the design and evaluation of early diagnosis programs contributing to global efforts for cancer control.
